# Carbon dioxide treatment modulates phosphatidic acid signaling and stress response to improve chilling tolerance and postharvest quality in paprika

**DOI:** 10.3389/fpls.2023.1287997

**Published:** 2023-11-16

**Authors:** Me-Hea Park, Kang-Mo Ku, Kyung-Ran Do, Hyang Lan Eum, Jae Han Cho, Pue Hee Park, Siva Kumar Malka

**Affiliations:** ^1^ Postharvest Research Division, National Institute of Horticultural and Herbal Science, Wanju, Republic of Korea; ^2^ Department of Plant Biotechnology, College of Life Sciences and Biotechnology, Korea University, Seoul, Republic of Korea; ^3^ Planning and Coordination Division, National Institute of Horticultural & Herbal Science, Wanju, Republic of Korea

**Keywords:** Capsicum annum L., chilling injury, membrane integrity, postharvest quality, lipid metabolism, phosphatidic acid, stress response, DREB

## Abstract

**Introduction:**

Paprika (*Capsicum annuum* L.) is prone to chilling injury (CI) during low-temperature storage. Although recent findings suggest that CO_2_ treatment may protect against CI, the effects of short-term CO_2_ treatment on CI and the underlying molecular mechanisms in paprika remain unknown. Therefore, this study aimed to examine the effect of short-term CO_2_ treatment on CI and postharvest quality in paprika during storage at cold storage and retail condition at physio-biochemical-molecular level.

**Methods:**

Paprika was treated with 20 and 30% CO_2_ for 3 h and stored at 4°C for 14 days, followed by additional storage for 2 days at 20°C (retail condition). Fruit quality parameters, including weight loss, firmness, color, and pitting were assessed, and the molecular mechanism of the treatment was elucidated using transcriptomic and metabolomic analyses.

**Results:**

Short-term treatment with 20 and 30% CO_2_ effectively maintained paprika quality during cold storage and retailer conditions, with reduced surface pitting, a common symptom of CI. Additionally, transcriptomic and metabolomic analyses revealed that 20% CO_2_ treatment induced genes associated with biosynthesis of phosphatidic acid (PA), diacylglycerol, triacylglycerol, and stress response, metabolites associated with phasphatidyl inositol signaling, inositol phosphate metabolism, and starch and sucrose metabolism.

**Conclusion:**

CO_2_ treatment activates PA biosynthesis through PLD and PLC-DGK pathways, and induces inositol phosphate, starch, and sucrose metabolism, thereby regulating chilling stress response via the ICE-CBF pathway. These findings suggest that short-term CO_2_ treatment enhances resistance to cold-induced injury and preserves postharvest quality in non-climacteric fruits, such as paprika, through activation of PA signaling, which improves membrane stability during cold storage and distribution.

## 1 Introduction

Postharvest storage and transportation of fresh produce are crucial stages in the supply chain that substantially affect product quality and shelf life. Among the various factors affecting the postharvest quality of fruits and vegetables, chilling injury (CI) remains a persistent challenge, particularly for chilling-sensitive crops, such as paprika (*Capsicum annuum* L.). CI is a physiological disorder characterized by the development of various symptoms, including tissue softening, water soaking, discoloration, and increased susceptibility to decay, leading to considerable economic losses for producers and retailers (Biswas et al., 2016; Park et al., 2021; Rai et al., 2022). Conventional approaches to mitigate CI often involve controlling storage temperatures above the chilling threshold, which is typically around 10°C for paprika (Lim et al., 2007). However, maintaining high temperatures during storage can result in accelerated deterioration and reduced shelf life (Rao et al., 2011). Therefore, it is critical to explore alternative strategies that can effectively alleviate CI, while preserving the quality attributes and extending the postharvest life of paprika.

Recently, carbon dioxide (CO_2_) treatment has emerged as a promising strategy for preserving the post-harvest quality of various horticultural products. For instance, continuous exposure to CO_2_ (5%) effectively maintained the postharvest quality of tomatoes during storage at 10°C (Taye et al., 2017). Similarly, strawberries exposed to 18% CO_2_ for 48 h prior to storage at 1°C exhibited enhanced resistance to softening and oxidative stress (del Olmo et al., 2022). Additionally, treatment with 95% CO_2_ for 36 h prior to storage at 1°C reduced the susceptibility of persimmons to CI (Besada et al., 2015). Moreover, treatment with 10% CO_2_ for 24 h in combination with modified atmosphere packaging effectively maintained the quality of sweet peppers stored at 10°C (Afolabi et al., 2023). Furthermore, treatment with 30% CO_2_ for 6 h prior to storage at 0°C reduced CI, extended storability, and preserved the sensory quality and antioxidant capacity of Madoka peach fruit (Tilahun et al., 2022). However, it is imperative to minimize the treatment duration to enhance the feasibility and cost-effectiveness of postharvest treatments for producers and distributors. Notably, studies have shown that exposing strawberries and tomatoes to 30% CO_2_ for only 3 h can effectively maintain quality and mitigate CI (Eum et al., 2021; Park et al., 2021).

The mechanism underlying CO_2_-induced postharvest quality preservation is attributed to its ability to reduce respiration rate and ethylene production. For instance, apples, melons, tomatoes, and bananas showed respiratory reduction following high CO_2_ treatment (Kubo et al., 1989; Park et al., 2021). CO_2_ pretreatment coupled with cold storage synergistically reduced ethylene production, leading to delayed ripening in tomatoes (Park et al., 2021). At the molecular level, ethylene biosynthesis and signaling genes are suppressed by CO_2_ pretreatment in tomatoes (Rothan et al., 1997; Park et al., 2021), and CO_2_ treatment can modulate genes encoding cell wall-degrading enzymes in strawberries (Eum et al., 2021). Additionally, CO_2_ pretreatment triggers the expression of genes involved in stress and the activity of antioxidant enzymes in several fruits and vegetables, including tomatoes, grapes, peaches, and strawberries (Rothan et al., 1997; Romero et al., 2016; Park et al., 2021; del Olmo et al., 2022; Tilahun et al., 2022). CI often disrupts membrane integrity due to altered fluidity and rigidity caused by temperature fluctuations during storage, resulting in cellular leakage, compromised physiological functions, and decrease in overall quality (Biswas et al., 2016; Valenzuela et al., 2017). Moreover, chilling stress can induce oxidative stress by triggering the production of reactive oxygen species (ROS) owing to disrupted electron transport chains and impaired antioxidant systems (Biswas et al., 2016; Valenzuela et al., 2017). Therefore, developing postharvest technologies that target membrane lipid metabolism and stress responses would be highly beneficial for inhibiting CI and maintaining the postharvest quality of fresh produce.

Despite the positive effects of short-term CO_2_ treatment in tomato and strawberry, its impact on the quality of stored paprika remains unexplored. Moreover, the molecular mechanisms of CO_2_-induced quality preservation and CI resistance remains unclear, particularly in non-climacteric fruits, such as paprika. Therefore, this study aimed to comprehensively evaluate the effect and molecular mechanism of short-term CO_2_ pretreatment on postharvest quality in paprika under cold storage and retail conditions, using transcriptomic and metabolomic analyses.

## 2 Materials and methods

### 2.1 Plant materials and treatments

Paprika fruits (cv. Sirocco, red color) were harvested at approximately 80–85% maturity stage. After arrival to the laboratory, the fruits were immediately treated with 20 and 30% CO_2_ (mixed with ambient air) or left untreated in a commercial cardboard box for 3 h in a closed chamber at room temperature (~20°C). After the treatment period, the chamber was flushed with air to remove CO_2_. In total, 30 boxes per treatment were used for the study, with each box containing 30 fruits. The CO_2_ concentration in the closed chamber was measured using a portable headspace analyzer (Dansensor, Ringsted, Denmark). Samples in the control group were flushed with ambient air, and the damaged fruits were discarded. The fruits were stored in a covered cardboard box at 4°C (cold storage) for 14 d or at 4°C for 14 d, followed by additional 2 d at 20°C (14 + 2 d; retail condition). Relative humidity was maintained at 90 ± 5% during the storage period.

### 2.2 Fruit quality evaluation

Briefly, 20 fruits were sampled per treatment for fruit quality assessment. The fruits were weighed to determine weight loss using an electronic weighing balance. Skin color was monitored using a color meter (Minolta CR-400; Konica Minolta, Osaka, Japan), and values were reported based on Hunter’s redness scale (a*). Firmness was analyzed using a texture analyzer (TA Plus Lloyd Instruments Ltd., Fareham, Hamshire, UK) equipped with a 5-mm plunger head (diameter) at a speed of 2 mm/s. Total soluble solid content (SSC) was analyzed using a digital refractometer (PAL-1, Atago Co. Ltd., Tokyo, Japan). Fruit pitting was expressed as the percentage of fruits that exhibited pitting. The final reported quality attributes were obtained from three independent replicates per treatment per day.

### 2.3 Light microscopy for tissue structure analysis

Tissue analysis was performed as previously described (Clément et al., 1996), with some modifications. Briefly, paprika tissues were fixed in 2.5% glutaraldehyde (v/v in a 0.1 M phosphate buffer) at pH of 7.2 with 4% sucrose (w/v) for 24 h. After three rinses with the above fixing buffer (30 min each), the samples were post-fixed with 1% OsO4 w/v in the same buffer with 4% sucrose (w/v) for 4 h. After rinsing three times (30 min each), the tissues were dehydrated in alcohol gradient series, transferred to propylene oxide, and embedded in Epon epoxy resin. Semi-thin sections (2.5 µm) were prepared using an ultra-microtome and placed on glass slides. The polysaccharide-specific reaction was performed using periodic acid-Schiff (PAS) and the tissue structures are shown in red. Sections for staining were first immersed in 1% periodic acid (w/v) for 30 min, followed by immersion in Schiff’s reagent for 40 min and in 5% sodium bisulfite (w/v) for 35 min. Thereafter, the sections were rinsed with distilled water, dried on a warm plate, and mounted on Histomount. The negative control was prepared by omitting the oxidation step using periodic acid. The samples were observed under a light microscope (Axioscop 2; Carl Zeiss, Germany).

### 2.4 Transcriptome analysis

Paprika fruits were sampled at days 0, 7, and 14 + 2 from the untreated control and 20% CO_2_-treated groups. Thereafter, five fruits were pooled from each sample, and the peel tissue was used for RNA isolation using the Qiagen RNA mini prep (Qiagen, USA). RNA purity and integrity were verified using an Agilent 2100 Bioanalyzer (Agilent Technologies, Santa Clara, CA, USA USA), and only RNA with an RNA integrity value (RIN) > 8 were used for library preparation. Library preparation and RNA sequencing (RNA-seq) were performed at C&K Genomics in Seoul, South Korea. The processed reads were aligned to the sequence of *Capsicum annuum* (AVRZ02) using HISAT v2.1.0 (Kim et al., 2015). Aligned reads were counted using featureCounts in the Subread package version 1.6.0100 (Liao et al., 2014). Count data were analyzed for differential gene expression using the EdgeR package (Robinson et al., 2010). The expression level of each transcript was normalized to the TMM (trimmed mean) using the M-value normalization method (Robinson and Oshlack, 2010). The filtered data were log2-transformed and subjected to quantile normalization. Differentially expressed genes (DEGs) were selected using *p* ≤ 0.05 and log2-fold change (FC) ≥ 1 as thresholds. Gene ontology (GO) enrichment sets of the DEGs were obtained using the DAVID (Databank for Annotation, Visualization, and Integrated Discovery) database (Dennis et al., 2003).

### 2.5 Quantitative real-time PCR

Quantitative real-time PCR (qRT-PCR) was performed as described by Park et al. (2021). Target genes were amplified on a CFX96 TouchTM Real-Time PCR Detection System (Bio-Rad, USA) using the iQTM SYBR Green Supermix (Bio-Rad) with specific primers (**Supplementary Table S1**). The qRT-PCR conditions were as follows: 95°C for 30 s, followed by 40 cycles of 95°C for 10 s and 55°C or 58°C for 40 s. The relative gene expression was calculated using the ΔΔCt method and normalized to that of the housekeeping genes *actin* and *elongation factor 1*. The qRT-PCR was performed using at least three biological replicates and two technical replicates.

### 2.6 Metabolome analysis using gas chromatography–mass spectrometry

Samples were prepared for primary metabolites profiling following previously described methods (Lisec et al., 2015; Song and Ku, 2021), with some modifications. Briefly, freeze-dried paprika powder was extracted in methanol. Ribitol and tetracosane were used as internal standards for water- and lipid-soluble compounds, respectively. Water- and lipid-soluble compounds were separated into two phases via liquid-to-liquid extraction using deionized water and chloroform, respectively. Each organic phase was fully dried using a SpeedVac. Thereafter, methoxyamide (in anhydrous pyridine) was added to a tube containing dried water-soluble phase and incubated at 37°C for 90 min under constant shaking at 800 rpm. For derivatization of the water-soluble metabolites, N-methyl-N-(trimethylsilyl)trifluoroacetamide and 1% trimethylchlorosilane (TMCS) were added, and the sample was incubated at 50°C for 20 min under constant shaking at 800 rpm. For derivatization of the lipid-soluble metabolites, N, O-bis (trimethylsilyl)trifluoroacetamide + TMCS was added to the sample. The mixture was incubated at 60°C for 60 min under constant shaking at 800 rpm. The sample was transferred to vials with an insert and 1 µL was injected into a gas chromatograph (Nexis GC-2030, Shimadzu, Kyoto, Japan) coupled to a gas chromatograph–mass spectrometer (GC/MS-QP 2020 NX, Shimadzu) and an autosampler with injector (AOC-20i PLUS, Shimadzu). Chromatographic separation was performed in a capillary column (DB-5MS, Agilent, CA, USA; 30 m × 0.25 mm coated with 0.25 µm film). The flow rate of the carrier gas (helium) was set to 1.2 mL·min^-1^. The mass spectrophotometry parameters were as follows: ion source temperature, interface temperature, and mass scan range were set to 300°C, 250°C, and 40-600 m/z, respectively. For analysis of water-soluble metabolites, the initial oven temperature was set at 80°C for 2 min, then increased to 330°C at a rate of 12°C·min^-1^, and maintained at 330°C for 5 min. For analysis of lipid-soluble metabolites, the initial oven temperature was set at 150°C for 1 min, then increased to 320°C at a rate of 12°C·min^-1^, and maintained at 320°C for 7 min. Metabolites were identified based on the library from National Institute of Standards and Technology (NIST) or standard compounds (**Supplementary Table S4**).

### 2.7 Statistical analyses

Data are presented as the mean ± standard error. Significant differences were determined using analysis of variance (ANOVA), followed by *t*-test for comparisons between groups. Partial least squares discriminant analysis (PLS–DA) and pathway analysis were performed using MetaboAnalyst (https://www.metaboanalyst.ca/). All analyses were performed using SAS v.9.2 (SAS Institute, Cary, NC, USA).

## 3 Results

### 3.1 CO_2_ treatment reduces chilling injury and maintains quality in paprika

**Figure 1:**
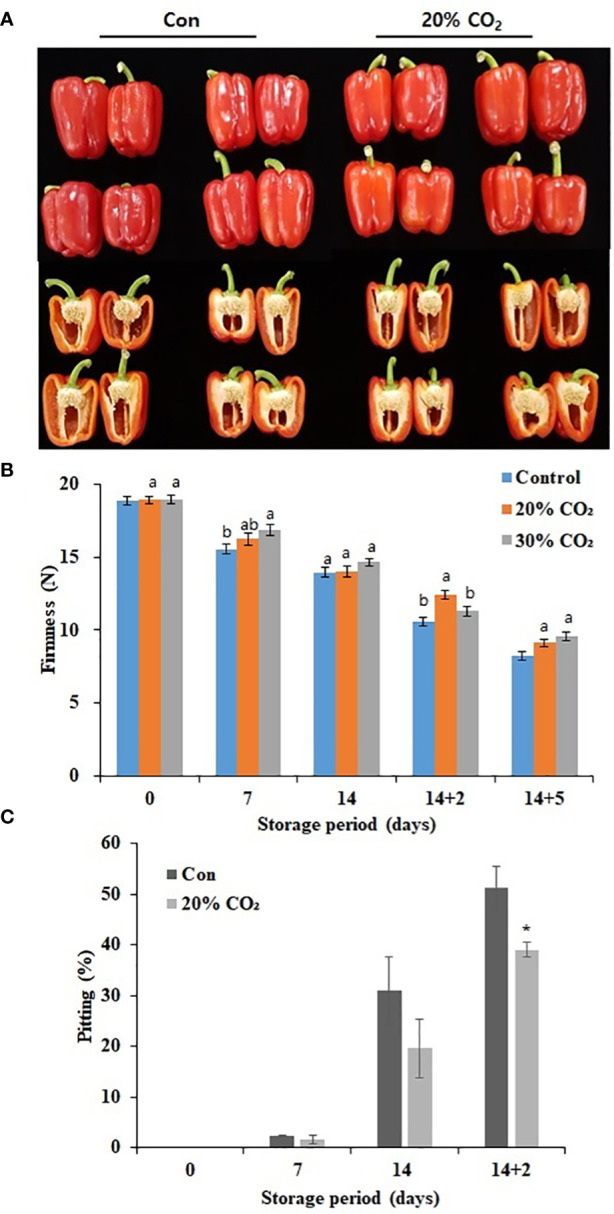
Effect of short-term CO_2_ treatment on postharvest quality and chilling injury in paprika. Representative images of CO_2_-treated and untreated fruits at 4°C for 14 days, followed by storage for 2 days at 20°C **(A)**, firmness **(B)**, and pitting **(C)** in paprika treated with CO_2_ and stored at 4°C for 14 days, and retail condition. Data represents the mean ± standard error of three replicates. At **(B)**, different letters on the graphs represent significant differences between the control and CO_2_ treatments (DMRT, P < 0.05). and at **(C)**, * represent t-test for comparisons between groups(p<0.1,**p < 0.05 and ***p < 0.0005).

Compared with that in the control group, treatment with 20 and 30% CO_2_ increased the respiration rate at day 0, indicating the successful absorption of CO_2_ in treated paprika (**Supplementary Figure S1**). However, there was a decrease in respiration rate during cold storage (4°C) for 14 days with respiration rate peaking at day 5 of storage at 20°C (**Supplementary Figure S1**). Notably, there was no significant difference in respiration rate between the treatment and control groups during storage at 4 and 20°C regardless of the CO_2_ treatment concentration. Additionally, treatment with 20% CO_2_ caused a decrease in hue value and fresh weight during cold storage (**Supplementary Table S2**). **Figure 1A** represents the images of CO_2_ -treated and untreated fruits stored at 14 days of cold storage at 4°C and additional 2 days of storage under retails condition at 20°C. Moreover, 20% CO_2_ - and 30% CO_2_ -treated paprika were significantly firmer than untreated fruits at 14 days cold storage at 4°C and 5 days at 20°C (**Figure 1B**; **Supplementary Table S2**). Specifically, fruits treated with 20% CO_2_ were 18.2% firmer than those in the control group after 14 days of cold storage and additional 2 days of storage under retails condition (14 + 2 days) (**Figure 1B**). There was no significant difference in SSC between CO_2_-treated and untreated fruits (**Supplementary Table S2**). The Pitting rate is a primary symptom of CI in paprika, fruits treated with 20 and 30% CO_2_ (**Supplementary Figure S3**) showed significantly lower surface pitting after transfer from cold storage to retail conditions. Specifically, only 39% of fruits treated with 20% CO_2_ showed surface pitting at day 2 after transfer from cold storage (14 days) to retail conditions compared with a rate of 51% in the control group (**Figure 1C**). CO_2_ treatment reduced the loss rate by about 12% during distribution, which is an economic benefit depending on the market price (**Supplementary Table S3**) Overall, these results suggest that CO_2_ treatment effectively delayed ripening and senescence and maintained fruit firmness during storage, which improved quality and reduced CI in paprika. As there were no notable differences between 20 and 30% CO_2_ treatments, 20% CO_2_ treatment was selected for further experiments.

### 3.2 CO_2_ treatment affects the transcriptome profile of paprika

**Figure 2:**
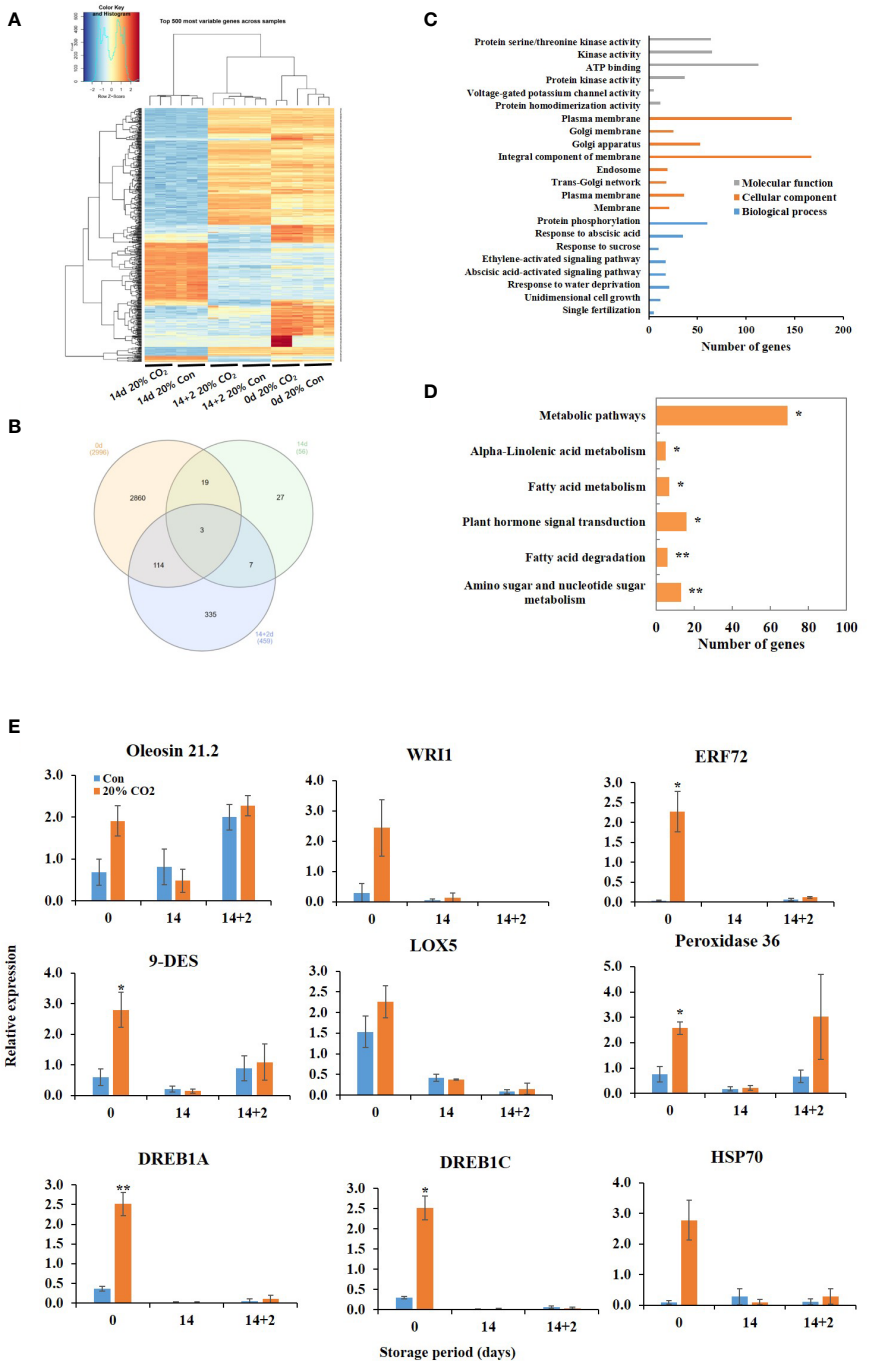
Transcriptome analysis of CO_2_-treated tomatoes. Heatmap **(A)** and Venn diagram **(B)** of differentially expressed genes (DEGs); Gene ontology functional categorization of DEGs **(C)**; KEGG pathway enrichment analysis of DEGs **(D)**; quantitative real-time PCR validation of lipid metabolism- and stress-related genes **(E)** in paprika treated with CO_2_ and stored at 4°C for 14 days, followed by storage for 2 days at 20°C (14 + 2). *p < 0.1, and **p < 0.01.

RNA sequencing was performed at days 0, 14, and 14 + 2 after CO_2_ treatment using pericarp tissues. Heatmap revealed remarkable changes in the transcriptome of the fruits following 20% CO_2_ treatment (**Figure 2A**). Differential expression analysis identified 3,511 DEGs in the treated vs. untreated groups, among which 2,996 DEGs were expressed at day 0, 56 DEGs at day 14, and 459 DEGs at day 14 + 2 after CO_2_ treatment (**Figure 2B**). GO functional annotation showed that the DEGs were enriched in different functional terms in the cellular components, biological processes, and molecular functions categories (**Figure 2C**). Kyoto Encyclopedia of Genes and Genomes (KEGG) enrichment analysis showed that the DEGs were enriched in amino sugar and nucleotide sugar metabolism, fatty acid degradation, fatty acid metabolism, alpha-linoleic acid metabolism, plant hormone signal transduction, and metabolic pathways (**Figure 2D**). All upregulated and downregulated genes in response to CO_2_ treatment are shown in **Tables S5**, **S6**, **S7**.

Membrane lipid metabolism plays an important role in the cold stress response. DEGs involved in lipid processes, such as phospholipases (*phospholipase D delta* and *phospholipase A1-II 1*), *diacylglycerol kinase 5* (*DGK5*), *diacylglycerol O-acyltransferase 1* (*DGAT1), omega-6 fatty acid desaturase* (*FAD*), *GDSL esterase/lipases* (*GDSL esterase/lipase At5g18430*, *GDSL esterase/lipase At1g71250*), *non-specific lipid-transfer proteins*, *oleosins (oleosin 21.2 kDa*, *oleosin 18 kDa*, *oleosin 18.5 kDa*), and *WRI1* were upregulated at day 0 (**Table 1**). Additionally, DEGs encoding the lipid-body membrane proteins, including *oleosins* (*oleosin 21.2 kDa*, *oleosin 18 kDa*, *oleosin 18.5 kDa*), were strongly induced at day 0. Moreover, DEGs encoding *glycerol-3-phosphate acyltransferase 3*, *FAD*, *enoyl-CoA delta isomerase 1*, and *lipid phosphate phosphatase 3* (*LPP3*) were upregulated in the CO_2_-treated fruits at day 14 + 2 (**Table 1**). At day 14, only 18 DEGs showed expression of more than 1.5 fold, and where mainly involved in encoding unknown proteins, except for cardiolipin synthase, which was downregulated. Additionally, DEG related to lipid-derived molecules, including oxylipins and jasmonates (*alpha-dioxygenase 1, 9-divinyl ether synthase*, *linolenate hydroperoxide lyase*, and *linoleate 9S-lipoxygenase*), were induced by CO_2_ treatment at days 0 and 14 + 2 after treatment (**Table 1**).

**Table 1:** Differentially expressed genes (DEGs) involved in lipid processes in paprika treated with CO_2._.

Gene ID	Annotation	Fold change (log 2 ratio)
0 d		
CA.PGAv.1.6.scaffold1148.6	Oleosin 21.2 kDa	13.43
CA.PGAv.1.6.scaffold206.50	Alpha-dioxygenase 1	10.77
CA.PGAv.1.6.scaffold1716.8	9-divinyl ether synthase	9.74
CA.PGAv.1.6.scaffold338.13	Oleosin 18 kDa	8.69
CA.PGAv.1.6.scaffold1856.8	Oleosin 18.5 kDa	8.42
CA.PGAv.1.6.scaffold1361.7	Short-chain dehydrogenase reductase 4	7.81
CA.PGAv.1.6.scaffold174.20	Oleosin 1	7.71
CA.PGAv.1.6.scaffold338.15	Oleosin 5	7.68
CA.PGAv.1.6.scaffold1344.22	AP2-like ethylene-responsive transcription factor AIL6	7.43
CA.PGAv.1.6.scaffold386.6	Poly [ADP-ribose] polymerase 3	7.37
CA.PGAv.1.6.scaffold134.49	Ethylene-responsive transcription factor WRI1	6.73
CA.PGAv.1.6.scaffold732.20	11-beta-hydroxysteroid dehydrogenase 1A	6.43
CA.PGAv.1.6.scaffold4.13	GDSL esterase/lipase At5g18430	6.01
CA.PGAv.1.6.scaffold781.9	GDSL esterase/lipase At1g71250	5.74
CA.PGAv.1.6.scaffold712.2	AP2-like ethylene-responsive transcription factor AIL6	5.58
CA.PGAv.1.6.scaffold508.5	Diacylglycerol kinase 5-like isoform X1	5.14
CA.PGAv.1.6.scaffold644.44	Putative phospholipid:diacylglycerol acyltransferase 2	5.00
CA.PGAv.1.6.scaffold1529.2	Peroxisomal fatty acid beta-oxidation multifunctional protein AIM1-like	4.72
CA.PGAv.1.6.scaffold508.7	Diacylglycerol kinase 5-like isoform X1	4.53
CA.PGAv.1.6.scaffold345.6	Non-specific lipid-transfer protein 2	4.34
CA.PGAv.1.6.scaffold508.6	Diacylglycerol kinase 5-like isoform X1	4.23
CA.PGAv.1.6.scaffold798.35	Phospholipase D delta-like	2.33
CA.PGAv.1.6.scaffold290.8	Linolenate hydroperoxide lyase, chloroplastic	1.77
CA.PGAv.1.6.scaffold1583.4	Diacylglycerol O-acyltransferase 1-like	1.58
CA.PGAv.1.6.scaffold702.15	Phospholipase A1-II 1-like	1.58
CA.PGAv.1.6.scaffold62.12	Phospholipase A1-Ibeta2, chloroplastic	1.02
14 d		
CA.PGAv.1.6.scaffold3104.1	Cardiolipin synthase (CMP-forming), mitochondrial	-2.22
14 + 2		
CA.PGAv.1.6.scaffold1152.14	Probable linoleate 9S-lipoxygenase 5	5.46
CA.PGAv.1.6.scaffold338.55	Glycerol-3-phosphate acyltransferase 3-like isoform X1	5.36
CA.PGAv.1.6.scaffold869.25	Enoyl-CoA delta isomerase 1, peroxisomal	5.21
CA.PGAv.1.6.scaffold575.39	Omega-6 fatty acid desaturase, endoplasmic reticulum	4.94
CA.PGAv.1.6.scaffold575.39	Omega-6 fatty acid desaturase, endoplasmic reticulum	4.94
CA.PGAv.1.6.scaffold1235.27	Lysine histidine transporter-like 8	4.00
CA.PGAv.1.6.scaffold575.29	Omega-6 fatty acid desaturase, endoplasmic reticulum	2.44
CA.PGAv.1.6.scaffold610.20	Lipid phosphate phosphatase 3, chloroplastic-like	2.09
CA.PGAv.1.6.scaffold103.21	Omega-6 fatty acid desaturase, endoplasmic reticulum	1.58

Bold value means paprika treated with CO2 and stored at 4°C for 14 days (14d), followed by storage for 2 days at 20°C (14 + 2). Od means before storage after treatment.

Furthermore, CO_2_ treatment modulated the expression of various stress-related genes, including dehydration-responsive element-binding proteins (*DREB1A* and *DREB1C*), *MYBs*, *heat shock 70 kDa protein*, *peroxidase 36*, *proline-rich protein 4*, *desiccation-related protein*, and *pathogenesis-related proteins*, at days 0 and 14 + 2 (**Table 2**). However, one DEG encoding *NAC domain-containing protein 7* was suppressed at day 14 after CO_2_ treatment (**Table 2**). qRT-PCR was performed to confirm the effects of CO_2_ treatment on *oleosin*, *9-DES*, *LOX5*, *ERF72*, *WRI1*, *peroxidase 36*, *HSP70*, *DREB1A*, and *DREB1C.* There were no significant differences in the expression levels of *lipoxygenase*, *oleosin*, *WRI*, and *HSP70* between CO_2_-treated and untreated fruits (**Figure 2E**). Notably, these genes, except for *oleosin* and *peroxidase36*, were specifically induced in CO_2_-treated fruits at day 0 (**Figure 2E**).

**Table 2:** Differentially expressed genes (DEGs) involved in stress response in paprika treated with CO_2._.

Gene ID	Annotation	Fold change (log 2 ratio)
0 d		
CA.PGAv.1.6.scaffold1129.55	Pathogenesis-related protein 5	8.37
CA.PGAv.1.6.scaffold104.8	Proline-rich protein 4	8.12
CA.PGAv.1.6.scaffold793.22	1-aminocyclopropane-1-carboxylate oxidase homolog 2	8.07
CA.PGAv.1.6.scaffold305.57	Desiccation-related protein PCC13-62	7.57
CA.PGAv.1.6.scaffold165.2	Cytochrome P450 81E8	7.41
CA.PGAv.1.6.scaffold160.28	Heat shock 70 kDa protein	7.11
CA.PGAv.1.6.scaffold851.27	Dehydration-responsive element-binding protein 2C	6.94
CA.PGAv.1.6.scaffold1129.23	Transcription factor MYB3-like	6.94
CA.PGAv.1.6.scaffold1239.15	Growth-regulating factor 8	6.11
CA.PGAv.1.6.scaffold927.3	Peroxidase 36	5.86
CA.PGAv.1.6.scaffold48.44	Leucine-rich repeat receptor-like protein kinase TDR	5.74
CA.PGAv.1.6.scaffold610.67	Transcription factor MYB48-like	5.50
CA.PGAv.1.6.scaffold862.63	Ethylene-responsive transcription factor RAP2-3	5.48
CA.PGAv.1.6.scaffold1297.5	Laccase-16	5.17
CA.PGAv.1.6.scaffold352.24	Cytochrome P450 94B3	4.21
CA.PGAv.1.6.scaffold138.3	Transcription factor MYB82-like	3.92
CA.PGAv.1.6.scaffold26.31	Dehydration-responsive element-binding protein 1C	2.47
CA.PGAv.1.6.scaffold1711.13	Probable WRKY transcription factor 72-like	2.45
CA.PGAv.1.6.scaffold809.26	Transcription factor MYB86-like	2.30
CA.PGAv.1.6.scaffold548.14	Dehydration-responsive element-binding protein 1A	2.15
CA.PGAv.1.6.scaffold907.9	MYB family transcription factor family protein	-1.14
CA.PGAv.1.6.scaffold362.14	Transcription repressor MYB4-like	-1.18
CA.PGAv.1.6.scaffold492.68	NAC domain-containing protein 21/22-like	-1.32
14 d		
CA.PGAv.1.6.scaffold330.12	NAC domain-containing protein 7	-2.69
14 + 2		
CA.PGAv.1.6.scaffold981.3	Thaumatin-like protein-like	5.49
CA.PGAv.1.6.scaffold2357.1	5-epiaristolochene synthase	3.67
CA.PGAv.1.6.scaffold566.15	Pathogenesis-related protein STH-21	3.60
CA.PGAv.1.6.scaffold1495.1	4-coumarate–CoA ligase 1	3.11
CA.PGAv.1.6.scaffold134.86	Pathogenesis-related protein PR-4B	3.02

Bold value means paprika treated with CO2 and stored at 4°C for 14 days (14d), followed by storage for 2 days at 20°C (14 + 2). Od means before storage after treatment.

### 3.3 CO_2_ treatment affects the metabolome of paprika

**Figure 3:**
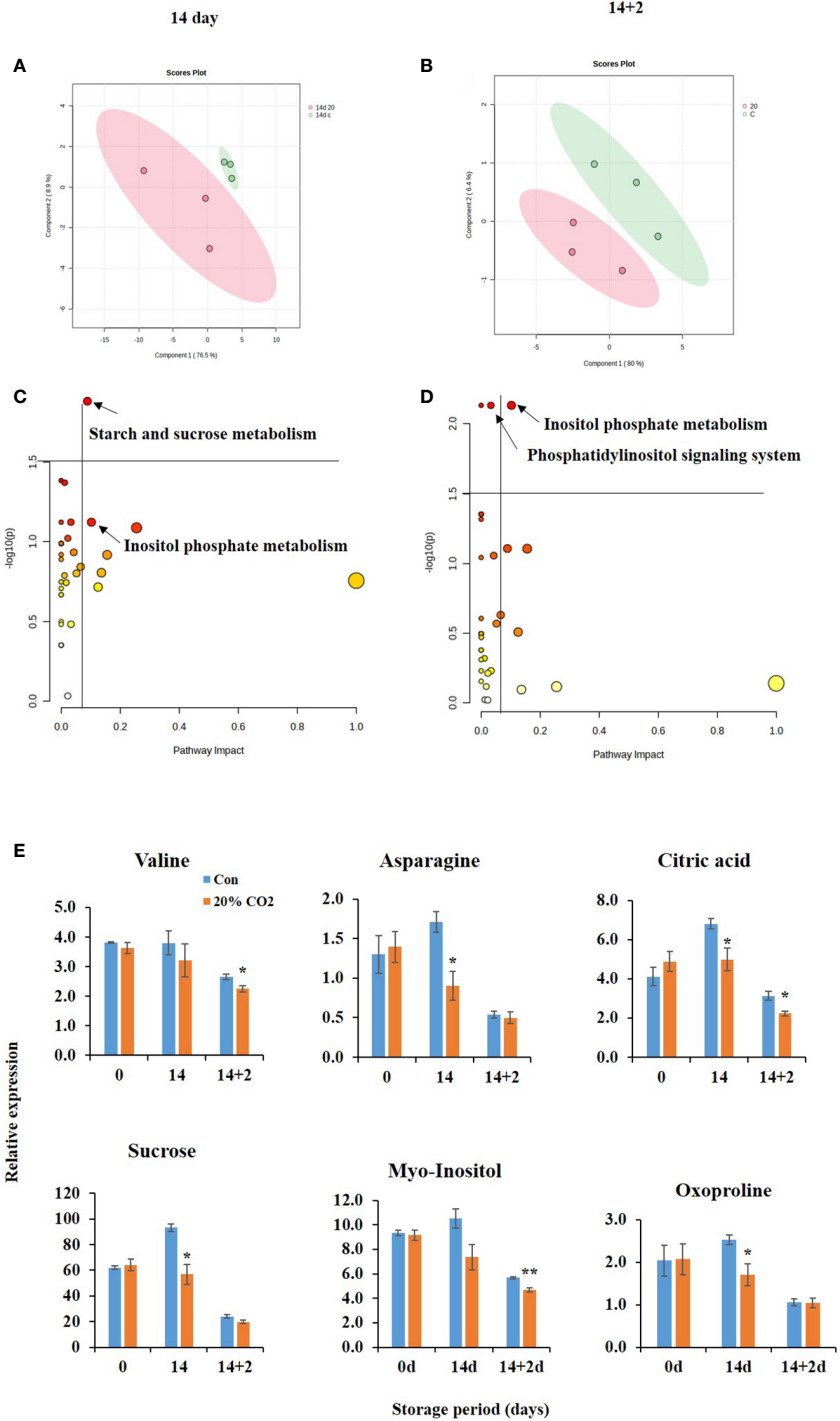
Metabolic changes in of CO_2_-treated paprika. Scores plot of partial least squares discriminant analysis of water soluble and lipid soluble metabolites at days 14 **(A)** and 14 + 2 **(B)**; Pathway analysis of water soluble and lipid soluble metabolites on days 14 **(C)** and 14 + 2 **(D)**; differential accumulation of metabolites **(E)** in paprika treated with CO_2_ and stored at 4°C for 14 days, followed by storage for 2 days at 20°C (14 + 2). *p < 0.1, and **p < 0.01.

The metabolite profiles of CO_2_-treated and untreated fruits were analyzed at days 0, 14, and 14 + 2 after CO_2_ treatment. In total, 36 metabolites, including 28 water-soluble and 8 lipid-soluble metabolites including 2 internal standards, were identified (**Supplementary Table S4**). PLS-DA was conducted to explore the effect of CO_2_ and the relationship between the metabolites. The two PLS-DA components collectively accounted for 85.4 and 86.4% of the total variance in the dataset at days 14 and 14 + 2, respectively (**Figures 3A, B**). Additionally, there was a clear separation of the two clusters, indicating the significant impact of CO_2_ on metabolites in paprika during storage (**Figures 3A, B**).

Further analysis indicated changes in pathways analysis at days 14 and 14 + 2 after CO_2_ treatment (**Figures 3C, D**). At day 14, the most significantly affected pathway was starch and sucrose metabolism’ pathway. At day 14 + 2, the most significantly affected pathway was the phosphatidylinositol signaling system and inositol phosphate metabolism. Furthermore, CO_2_ treatment significantly reduced the levels of the metabolites valine, asparagine, citric acid, sucrose, myoinositol, and oxyproline at days 14 and 14 + 2 after CO_2_ treatment (**Figure 3E**), indicating potential alteration of the TCA cycle, electron transport chain, and stress tolerance mechanisms in CO_2_-treated fruits. Decreased citric acid and sucrose levels in CO_2_-treated fruits (**Figure 3E**) suggest their channelizing into GABA shunt pathway.

### 3.4 Anatomical analysis of pericarp of paprika treated with CO_2_

**Figure 4:**
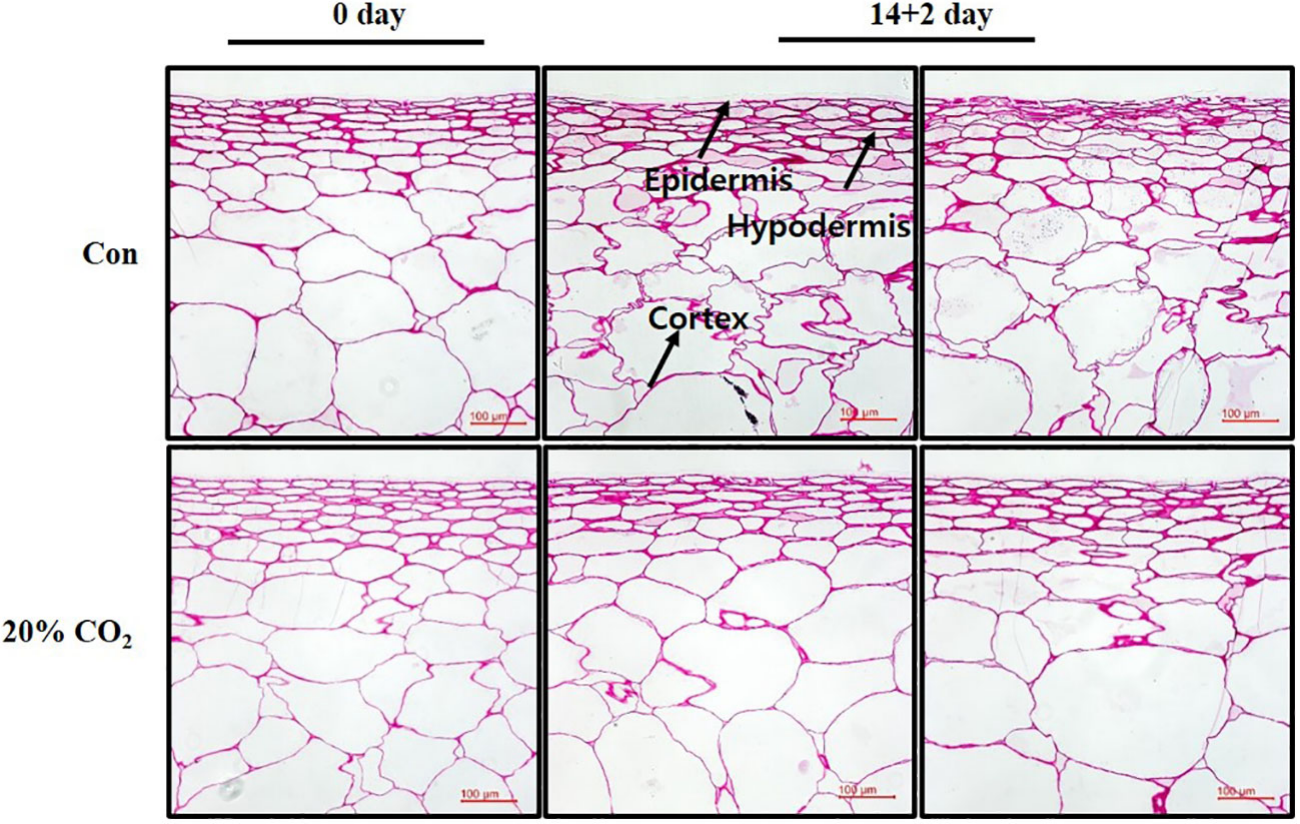
Microscopic analysis of pericarp tissues of paprika.

In this study, surface pitting was observed on the fruits at day 14 + 2 of storage (**Figure 1C**). Microscopic examination of cross-sections of pericarp tissues of the fruits at days 0 and 14 + 2 showed that the epidermis and hypodermis of the pericarp tissues appeared to have a compact cell size and shrinking cell morphology, suggesting severe water loss in the hypodermal layer (**Figure 4**). Notably, hypodermal cells were substantially shirked and appeared to collapse compared with epidermal cells (**Figure 4**), suggesting cell membrane impairment in hypodermal cells during long-term low-temperature storage.

## 4 Discussion

### 4.1 Short-term CO_2_ treatment enhances postharvest quality and reduces CI in paprika

The primary CI symptoms in paprika such as surface pitting, calyx discoloration remains a major concern, leading to substantial economic losses. CI is initiated in fruits exposed to cold temperatures; however, the symptoms are more evident when the fruits are shifted from cold storage temperatures to non-chilling temperatures (Biswas et al., 2016). In the present study, short-term treatment with 20 and 30% CO_2_ for 3 h prior to cold storage delayed ripening progression, enhanced firmness, reduced weight loss, and minimized surface pitting (**Figure 1**), which was consistent with previous findings in CO_2_-treated crops, including tomatoes, strawberries, persimmons, peaches, and sweet peppers (Besada et al., 2015; Eum et al., 2021; Park et al., 2021; Tilahun et al., 2022; Afolabi et al., 2023). Notably, our study differs from previous approaches that utilized longer treatment durations, ranging from 6 to 48 h or continuous. Although the effect of CO_2_ treatment for 3 h on tomato and strawberries has been previously examined, this is the first study to best of our knowledge to examine the effects of short-term CO_2_ treatment on postharvest quality and CI in paprika. The results of the present study are attributed to the ability of CO_2_ to modulate respiration rates and ethylene production, as observed in studies on tomatoes and other fruits (Kubo et al., 1989; Taye et al., 2017; Park et al., 2021; Tilahun et al., 2022).

### 4.2 Short-term CO_2_ treatment activates genes associated with phosphatidic acid biosynthesis and stress response

Transcriptomic and metabolomic analyses revealed the intricate molecular responses triggered by CO_2_ treatment in paprika. Specifically, CO_2_ treatment activated specific DEGs and metabolites associated with lipid processes and stress responses, shedding light on the underlying mechanisms. Particularly, CO_2_ treatment activated genes involved in phosphatidic acid (PA) biosynthesis, a central precursor for glycerophospholipids, galactolipids, and triacylglycerol (TAG) biosynthesis; moreover, PA plays a pivotal role in cellular responses to stress conditions (Hong et al., 2016; Perlikowski et al., 2016; Yu et al., 2019; Wu et al., 2022). PA is produced through the acylation of lysophosphatidic acid (LPA), which is derived from glycerol 3-phosphate by the enzyme glycerol 3-phosphate acyltransferase (GPAT) (Nakamura, 2017). PA biosynthesis via acylation steps is the start of the glycerolipid *de novo* biosynthesis. In this study, CO_2_ treatment induced the expression of *GPAT* (**Table 1**), indicating the induction of PA production in these fruits. Alternatively, PA levels are controlled by phospholipase (PL) D and PLC-DGK (diacylglycerol kinase) pathways, involving PLs, phosphates, and lipid kinases (Wu et al., 2022). In the PLD pathway, the structural phospholipids are hydrolyzed by PLD to produce PA and soluble head groups (Wu et al., 2022). In the PLC-DGK pathway, PLC acts on phosphatidylinositol 4,5-bisphosphate (PtdInsP2) to produce DAG and inositol phosphate (IP) 3. Notably, DAG can be phosphorylated by DGK to form PA (Wu et al., 2022). Additionally, PA can be dephosphorylated back into DAG by lipid phosphate phosphatases (LPP) (Craddock et al., 2017; Su et al., 2021). In this study, CO_2_ treatment enhanced the expression of *PLDδ*, *DGK5s*, and *LPP3*, indicating the activation of PLD and PLC-DGK pathways. These pathways are the two principal routes that produce signaling PA and have been extensively studied for their early response to cold stress (Vergnolle et al., 2005; Wu et al., 2022). For instance, *PLDs* and *DGKs* were responsive to low temperature in peppers (Kong et al., 2019). Deactivating *PLDδ* makes Arabidopsis plants more sensitive to freezing, while its overexpression enhances freezing tolerance (Li et al., 2004). Furthermore, DAG can serve as a substrate for the synthesis of various lipids, including membrane phospholipids and TAGs. Diacylglycerol O-acyltransferase 1 (DGAT) transfers a fatty acyl group from a fatty acyl-CoA molecule to a DAG molecule, resulting in the formation of TAG (Wu et al., 2022). In this study, the expression of DEGs encoding DGAT and oleosins, the structural proteins of TAGs, were induced in the CO_2_-treated fruits (**Table 1**; Shimada et al., 2008). The *dgat1* mutant lines exhibited reduced cold tolerance, and DAG and PA levels were significantly increased in Arabidopsis (Tan et al., 2018). Moreover, the dynamic balance of PA, DAG, and TAG is an important protective strategy to combat freezing temperatures (Tan et al., 2018).

The ICE–CBF/DREB1 transcriptional cascade has been extensively studied for its role in cold signaling (Chinnusamy et al., 2007). *ICE1* transcription factor directly activates cold-responsive genes by binding to cis-elements in the *CBF3/DREB1a* and *CBF2* (*DREB1C*) promoters (Chinnusamy et al., 2007). In the present study, *DREB1A* and *DREB1C* were expressed at day 0 after CO_2_ treatment (**Table 2**; **Figure 2E**), indicating the potent impact of CO_2_ treatment on the early activation of crucial components of the ICE–CBF/DREB1 pathway. Moreover, PLC and PLD pathways function upstream of the ICE–CBF/DREB1 pathway (Vergnolle et al., 2005). These results indicate that CO_2_ treatment may trigger ICE–CBF/DREB1 pathway to regulate chilling stress. Furthermore, the jasmonate signaling pathway acts as a pivotal upstream regulator of the ICE–CBF/DREB1 pathway, and plays a pivotal role in enhancing freezing tolerance in Arabidopsis (Hu et al., 2013). Remarkably, CO_2_ treatment induced the expression of jasmonate synthesis-related genes, specifically *alpha-dioxygenase 1*, *9-divinyl ether synthase* (*DES)*, *9S-lipoxygenase* (*LOX5*), in the treated fruits (**Table 1**; **Figure 2E**). The concurrent induction of *DREBs* and jasmonic acid biosynthesis-related genes strongly suggests the activation of the ICE–CBF pathway following CO_2_ treatment. Moreover, CO_2_ treatment triggered the expression of known stress-responsive genes, such as *peroxidase 32*, *heatshok protein 70*, *pathogenesis related proteins* (**Table 2**; **Figure 2E**), and promoted stress defense mechanisms. The upregulation of stress-related genes may have amplified antioxidant enzyme activity, as confirmed by ABTS and DPPH assays, and enhanced polyphenol content (**Supplementary Figure S2**). Overall, these results indicate that CO_2_ enhances resistance to freezing stress in fruits through a multifaceted approach, thereby improving postharvest quality.

Intricate stress signaling might induce lipid processes, as evidenced by enhanced expression of the membrane integrity associated gene *fatty acid desaturases* (*FADs*) in CO_2_-treated fruits (**Table 1**; **Figure 2E**), leading to improved membrane stability (**Figure 4**), which may contribute to extending the shelf life of the fruits. This is of particular significance, considering that the balance of unsaturated fatty acids within the lipid bilayer plays a pivotal role in plant responses to CI (Zhang and Tian, 2010). Moreover, heterologous overexpression of *Eriobotrya japonica’s FAD8* in Arabidopsis increased the expression of *ICE*-*CBF*-*cold regulated genes* in response to low temperatures (Xu et al., 2023), indicating intricate interaction between lipid process and stress signaling during chilling stress.

### 4.3 Short-term CO_2_ treatment activates metabolites associated with inositol phosphate metabolism and starch and sucrose metabolism

At day 14 of cold storage, starch and sucrose metabolism were remarkably altered, suggesting that high CO_2_ exposure may play a role in modulating carbohydrate metabolism in CO_2_-treated paprika during cold storage. Starch and sucrose, the principal carbohydrates in plants, play critical roles in energy storage and transfer (Zhu et al., 2023), and alterations in this pathway indicates that short-term CO_2_ treatment increases the tricarboxylic acid (TCA) cycle, potentially affecting fruit respiration. Exposing agricultural produce to elevated levels of CO_2_, especially for short durations may promote anaerobic metabolism or stress responses. Under these conditions, a reduction in mitochondrial respiration, which in turn limits the availability of ATP for energy-demanding processes. In response to this energy crisis, fruit metabolism adapts by enhancing substrate-level ATP production. This adaptation involves various processes, including the breakdown of soluble sugars and the degradation of starch (Gorin et al., 1978; Planchet et al., 2017; Brizzolara et al., 2020).

Terzoudis et al. (2022) reported that a decrease in oxygen levels was associated with reduced levels of sucrose, citrate, and valine in postharvest peach fruits. Furthermore, under anaerobic conditions, certain amino acids, including valine, can be metabolized via the branched-chain amino acid degradation pathway to produce compounds that feed into the TCA cycle or the electron transport chain (Araújo et al., 2011). This might lead to a decrease in valine levels in CO_2_-treated fruits (**Figure 3E**). Another possible explanation is that a sudden elevation in CO_2_ may affect protein synthesis and degradation in cells (Brizzolara et al., 2020). Notably, Tanou et al. (2017) reported differential accumulation of valine was associated with CI tolerance in peach fruits. Similarly, the reduction in citric acid and sucrose levels can be interpreted as continuous metabolism under elevated CO_2_ levels. Although it is known that high levels of CO_2_ and low O_2_ inhibit cell respiration (Kubo et al., 1989), metabolism might increase through the GABA shunt pathway under low O_2_ conditions (Li et al., 2021).

IPs and phosphatidylinositol (PI) signaling pathways are involved in several biological processes, including chilling stress. IP3 is generated by PLC-mediated hydrolysis of PtdInsP2, and triggers a set of cellular processes by releasing calcium, which acts as a secondary messenger to transduce cold signals (Sun et al., 2021). In this study, inositol phosphate metabolism was identified at both days 14 and 14 + 2, emphasizing its importance and potential sensitivity to CO_2_ exposure. Phosphatidylinositol signaling is pivotal for various cellular processes, including cell growth, differentiation, and motility (Sun et al., 2021). The phosphatidylinositol signaling system and inositol phosphate metabolic pathways are linked to freezing tolerance and CI regulation in plants (Zakharian et al., 2010; Sun et al., 2021). Notably, metabolite changes in CO_2_-treated fruits were associated with phosphatidylinositol signaling and inositol phosphate metabolism (**Figure 3D**), suggesting their potential role in CO_2_-induced chilling tolerance in paprika.

Notably, the cellular response to CO_2_ in non-climacteric fruits appears to differ from that in climacteric fruits. For instance, treatment with 30% CO_2_ for 3 h maintained the quality of tomatoes and protected against CI through associated with transcriptional changes in ethylene-related genes and respiratory-related metabolism (Park et al., 2021), whereas CO_2_ treatment maintained the quality of paprika, a non-climacteric fruit, by modulating mainly lipid-related processes, stress responses, and metabolism of starch and inositol phosphates.

## 5 Conclusion

**Figure 5:**
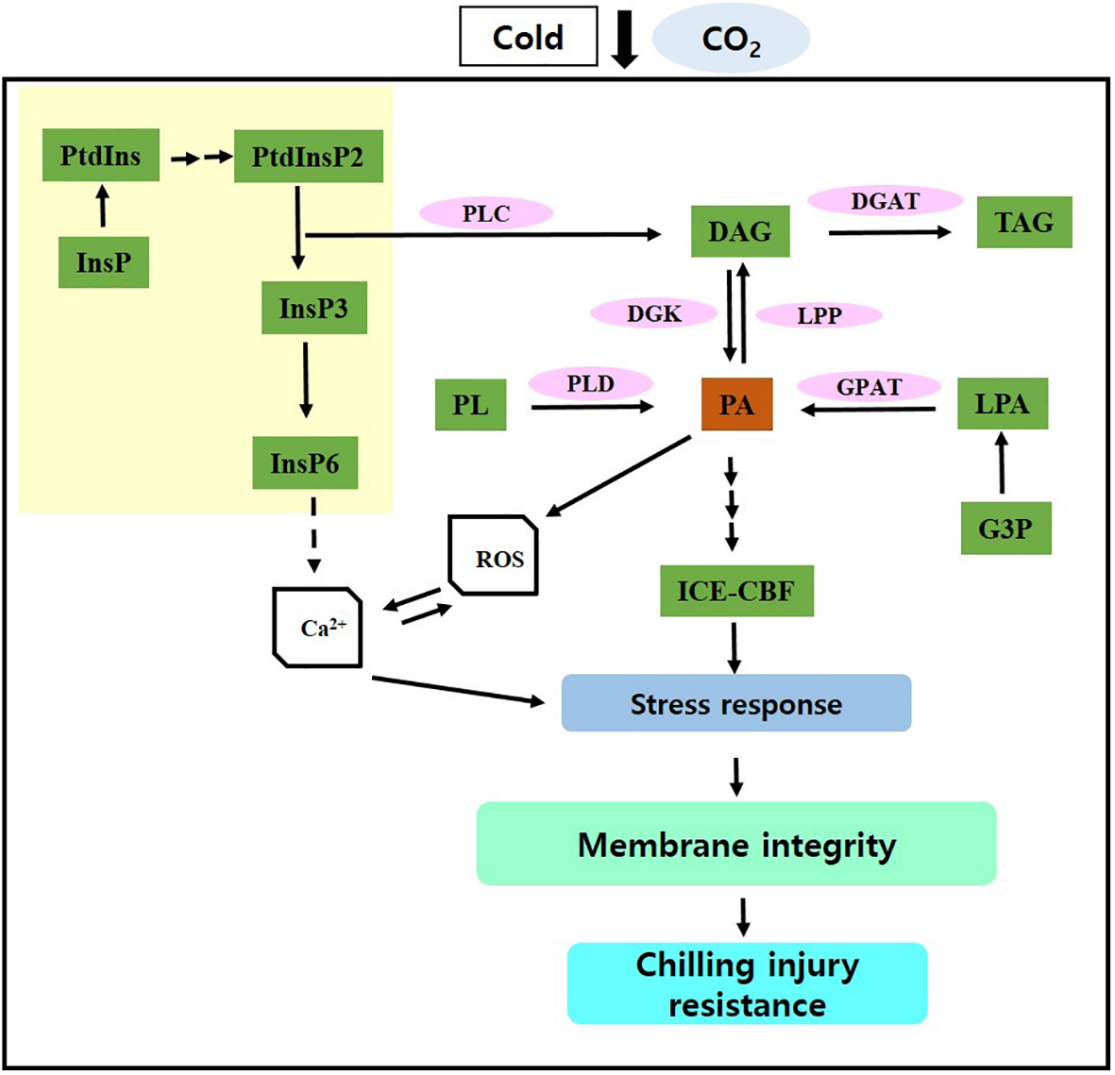
Potential molecular mechanism through which CO_2_ maintains postharvest quality and protects against chilling injury. InsP, inositol phosphate; PtdIns, phosphatidylinositol; PtdInsP, phosphatidylinositol phosphate; PL, phospholipid; PLD, phospholipase D; PLC, phospholipase C; PA, phosphatidic acid; DAG, diacylglycerol; TAG, triacylglycerol; G3P, glycerol-3-phosphate, LPA, lysophosphatidic acid; DGK, DAG kinase; GPAT, G3P acyltransferase; LPP, LPA phosphatase. Solid arrows represent established pathways; dashed arrows indicate potential pathways; array of arrows indicate multiple steps; yellow region indicate inositol phosphate metabolism.

Short-term CO_2_ treatment reduced CI and improved postharvest quality in paprika by activating PA synthesis and its signaling via the PLD and PLC–DK pathways, inducing stress signaling via the ICE–CBF pathway, and enhancing lipid processes and antioxidant defense mechanisms, thereby promoting membrane stability (**Figure 5**). Overall, these findings contribute to the advancement of innovative strategies for preserving postharvest quality in paprika and minimizing losses. However, further studies are necessary to examine the effects of short-term CO_2_ treatment on the postharvest quality of other crop species under cold storage.

## Data availability statement

The datasets presented in this study can be found in online repositories. The names of the repository/repositories and accession number(s) can be found here: https://www.ncbi.nlm.nih.gov/bioproject/PRJNA1019830/.

## Author contributions

M-HP: Conceptualization, Funding acquisition, Methodology, Project administration, Resources, Supervision, Validation, Visualization, Writing – review & editing. KK: Data curation, Methodology, Supervision, Writing – original draft. K-RD: Data curation, Writing – original draft. HE: Data curation, Software, Writing – original draft. JC: Data curation, Methodology, Writing – original draft. PP: Data curation, Investigation, Resources, Writing – original draft. SM: Conceptualization, Data curation, Formal Analysis, Investigation, Methodology, Software, Supervision, Validation, Visualization, Writing – original draft, Writing – review & editing.
