# Supplementary material for: Carbon dioxide treatment modulates phosphatidic acid signaling and stress response to improve chilling tolerance and postharvest quality in paprika
Source: Front Plant Sci. 2023 Nov 16;14:1287997. doi: 10.3389/fpls.2023.1287997 (PMC10711834; doi:10.3389/fpls.2023.1287997)
Supplement: Supplementary file 1 [file DataSheet_1.zip › Supplementary Figures 1-3 and Tables 1-4.docx]

Supplementary Material

# Supplementary Methods

## Gas chromatography analysis

Respiration rate and ethylene production were analyzed using gas chromatography (Agilent 7890B-GC; Agilent Technologies, USA). Briefly, 1 mL of gas was sampled from the boxes for 2 h. The injection and column temperatures were 110 °C and 70 °C, respectively. The thermal conductivity detector and flame ionization detector used for the CO_2_ and ethylene measurements were set at 150 °C and 250 °C, respectively.

## Antioxidant analysis

Sample extraction was performed following the procedure described by Vasco et al. (2008) with slight modifications. Briefly, freeze-dried fruit powder (500 mg) was extracted twice at 25 ± 2 °C for 2 h under constant shaking, first with a 20-mL mixture of methanol and water (50:50 v/v) and then with a 20-mL mixture of acetone and water (70:30, v/v). Thereafter, the extracts were centrifuged at 4000 rpm for 15 min to remove the supernatant, and the filtered extracts were used to assess the total phenolic and antioxidant activities. All reagents were purchased from Sigma–Aldrich (St. Louis, MO, USA). Total polyphenol content was measured using a modified version of the method described by Singleton and Rossi (1965). Briefly, 100 µL of extract or standard was reacted with 100 µL of 1 N Folin–Ciocalteu reagent for 3 min, followed by the addition of 1 mL of 2% sodium carbonate solution. The mixture was incubated for 30 min and the absorbance was read at 726 nm using a microplate reader (EPOCH2, BioTek Instruments Inc., Winooski, VT, USA). The results are expressed as gallic acid (GAE) equivalents (GAE, mg/g DW).

1,1-Diphenyl-2-picrylhydrazyl (DPPH) radical scavenging activity of the samples was assessed using the method described by Dietz et al. (2005). Briefly, 20 µL of extract was mixed with 180 µL of 0.18 mM DPPH, and then vortexed. After standing for 20 min, the absorbance was measured at 515 nm using a microplate reader. Methanol without DPPH radicals was used as the blank reference, and the absorbance was converted to DPPH radical scavenging activity. Additionally, 2,2-azino-bis-3-ethylbenzothiazoline-6-sulfonic acid (ABTS) radical-scavenging activity was evaluated according to the method developed by Re et al. (1999), with some modifications. Briefly, an ABTS stock solution (7 mM) was prepared from 7 mM ABTS and 2.45 mM potassium persulfate solution and kept in the dark for 16 h. The reagent was adjusted with ethanol until the absorbance at 734 nm reached 0.7 ± 0.02 at ambient temperature (25 ± 2 °C). Thereafter, 20 μL of each extract was mixed with 180 μL of the ABTS+ solution, and the absorbance at 734 nm was measured after 10 min at ambient temperature (25 ± 2 °C). The DPPH and ABTS radical scavenging activity were expressed as Trolox equivalent (TE) antioxidant capacity (μmol TE g/dw).

# Supplementary Figures
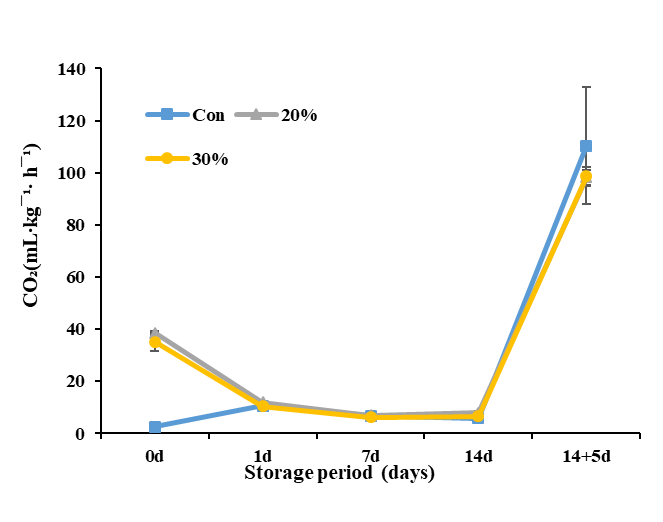


**Supplementary Figure 1**. Respiration rate in paprika treated with CO_2_ and stored at 4 °C for 14 days, followed by storage for an additional 2 days (14 + 5) at 20 °C.


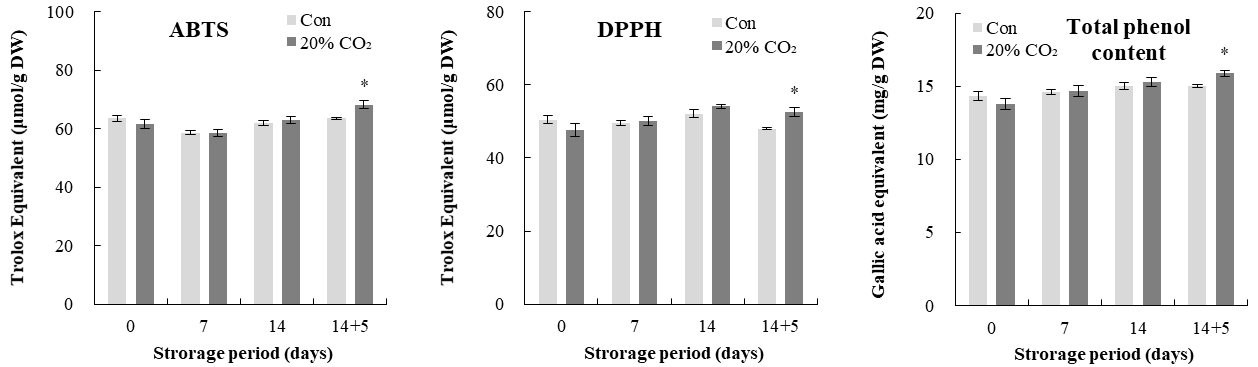


**Supplementary Figure 2**. Activity of antioxidant enzymes in paprika treated with CO_2_ and stored at 4 °C for 14 days, followed by storage for an additional 5 days (14 + 5) at 20 °C. Data are the mean ± standard error of three replicates. * represents p < 0.05 and ** represents p < 0.005.

**Supplementary Figure 3**. Effects of CO_2_ treatment on the pitting rate of paprika stored at 4 ℃ for 14 days and transferred to 20 ℃ for 2 days (14+2). Data are the mean ± standard error of three replicates. The different letters on the graphs represent significant differences between the control and CO_2_ treatments (DMRT, P < 0.05).

# Supplementary Tables

**Table S1.** Primer sequences for quantitative RT-PCR amplification.

| **Gene** | **Accession** | **Forward primer sequences (5'-3')** | **Reverse primer sequences (5'-3')** |
| --- | --- | --- | --- |
| ***Actin*** | *XM_016683691.2* | GGGATG GGTCAAAAGGATGC | GGTTCAAGGGTGCTTCAGTG |
| ***EF1*** | *LC164844.1* | TTCACTGCCCAGGTCATCAT | TGGCTTGGTGGGAATCATCT |
| ***Oleosin 21.2*** | CA.PGAv.1.6.scaffold1148.6 | TCAATTCACTATGGCTGACC | GCTAAACAACAGAAAGACGG |
| ***WRI1*** | CA.PGAv.1.6.scaffold134.49 | GGAAGTAAGGAACAATCCGT | TCAATCCTCCCAAGAATAGC |
| ***ERF72*** | CA.PGAv.1.6.scaffold862.63 | TCGAGAACAATGAACCCGTTATC | CCACGACCCTTCCATTCATATT |
| ***9-DES*** | CA.PGAv.1.6.scaffold1716.8 9 | GCACACTTTCCCTCTACCATAC | GGACCCAAGGAGTTGTAGAAAG |
| ***LOX5*** | CA.PGAv.1.6.scaffold1152.14 | GAGGAATGGGACAGGGTTTATG | AGCAGATCCTCCCAAGATAGT |
| ***Peroxidase 36*** | CA.PGAv.1.6.scaffold927.3 | GCTTTGTCAAGGGTTGTGATG | TCCACGTGCTGAATTCCTATT |
| ***DREB1A*** | CA.PGAv.1.6.scaffold548.14 | GTTCTCCTCAGACACCAGAAAG | CGTTAGTCCTTCCGCCATATT |
| ***DREB1C*** | CA.PGAv.1.6.scaffold26.31 | GTATAGCAGCTCATGTGGGATAG | ATCACTTTCCACGTCCTCATC |
| ***HSP70*** | CA.PGAv.1.6.scaffold160.28 | CTAAAGATGCTGGTGCGATTTC | CCTCTTCTCGACGCTTTCTTAT |

**Table S2.** Effect of CO_2_ treatment on quality (weight loss; firmness; SSC: soluble solid content) and color (Hue values) of paprika treated with CO_2_ and stored at 4 °C for 14 days, followed by storage for an additional 2 days (14+2) at 20 °C.

|  | **0 day** | **7 days** | **14 days** | **14+2 days** | **14+5 days** |
| --- | --- | --- | --- | --- | --- |
|  | **Weight loss (%)** | | | | |
| **Control** | 0.00±0.00 Ae | 1.06±0.04 Ad | 2.50±0.01 Ac | 4.33±0.17 Ab | 6.64±0.30 Aa |
| **20% CO₂** | 0.00±0.00 Ae | 0.89±0.01 Bd | 2.18±0.11 Bc | 4.13±0.05 Ab | 6.44±0.10 Aa |
| **30% CO₂** | 0.00±0.00 Ae | 1.02±0.08 ABd | 2.33±0.07 ABc | 4.32±0.08 Ab | 6.56±0.13 Aa |
|  | **Firmness (N)** | | | | |
| **Control** | 18.88±0.28 Aa | 15.56±0.32 Bb | 13.96±0.32 Ac | 10.59±0.30 Bd | 8.23±0.28 Be |
| **20% CO₂** | 18.92±0.24 Aa | 16.24±0.38 ABb | 14.01±0.34 Ac | 12.42±0.30 Ad | 9.13±0.27 Ae |
| **30% CO₂** | 18.94±0.28 Aa | 16.86±0.37 Ab | 14.66±0.27 Ac | 11.33±0.34 Bd | 9.58±0.30 Ae |
|  | **SSC (˚Brix)** | | | | |
| **Control** | 6.62±0.07 Ab | 6.56±0.07 Ab | 6.98±0.06 Aa | 7.08±0.06 Aa | 7.08±0.07 Aa |
| **20% CO₂** | 6.60±0.10 Ab | 6.68±0.05 Ab | 6.96±0.07 Aa | 6.96±0.02 Aa | 7.10±0.07 Aa |
| **30% CO₂** | 6.68±0.06 Ab | 6.74±0.05 Ab | 7.00±0.07 Aa | 7.06±0.05 Aa | 7.06±0.09 Aa |
|  | **H value** | | | | |
| **Control** | 38.13±0.60 Ba | 35.13±0.40 Ab | 35.39±0.44 Ab | 31.95±0.29 Ac | 29.27±0.21 Ad |
| **20% CO₂** | 38.51±0.71 Ba | 34.20±0.47 Ab | 33.83±0.38 Bb | 31.21±0.24 Ac | 28.41±0.16 Bd |
| **30% CO₂** | 41.15±0.99 Aa | 35.25±0.50 Ab | 35.50±0.56 Ab | 31.89±0.32 Ac | 29.41±0.24 Ad |

The same capital letter within each column or the same small letter within each row indicates non-significantly different means at p < 0.05, according to DMRT.

Table S3. Analysis of economic effects of carbon dioxide treatment on paprika

○ Economic analysis (unit: Korean won/1,000kg)

| Loss factor(A) | Earning factor(B) |
| --- | --- |
| o Variable cost  - CO₂ gas fee : 30,000 won  Total (A) : 30,000won | o Increased benefits (Based on 1,000kg)  - Loss rate during distribution (Pitting(%) : non-treated 51% vs CO_2_ treated 39% at 14 days cold storage and retail condition 2 days  = CO_2_ treatment 12 % benefit  If the price of paprika is 30,000 won per box(5kg) and there are 200 boxes(1000kg), benefit is  - 30,000 won/box(5kg), * 200 box * 12% = 900,000won  - Total(B) : 900,000won |
| o Estimated profit (B-A): 900,000 –30,000 = 870,000 won | |

**Table S4.** Identification of primary metabolites in paprika.

| No. | Compound | RT (min) | Characteristic ion (*m/z*) | Derivatization^a^ | Identification^b^ | Similarity | Remarks |
| --- | --- | --- | --- | --- | --- | --- | --- |
| 1 | 1,3-Propanediol | 4.888 | 73, 115, 147 | 2TMS | NIST | 93 | Water soluble |
| 2 | Oxalic acid | 5.847 | 73, 133, 147 | 2TMS | NIST | 82 | Water soluble |
| 3 | 1,4-Butanediol | 6.09 | 132, 116, 147 | 2TMS | NIST | 89 | Water soluble |
| 4 | Valine | 6.662 | 73, 103, 144 | 2TMS | NIST | 71 | Water soluble |
| 5 | Serine | 7.095 | 73, 116, 147, 219 | 2TMS | NIST | 87 | Water soluble |
| 6 | Butanedioic acid | 7.657 | 73, 172, 147 | 2TMS | NIST | 82 | Water soluble |
| 7 | Palmitic acid | 8.705 | 73, 117, 313 | TMS | NIST | 96 | Lipid soluble |
| 8 | Norvaline | 8.808 | 45, 147, 82 | TMS | NIST | 69 | Water soluble |
| 9 | Malic acid | 9.202 | 73, 147, 233 | 3TMS | NIST | 69 | Water soluble |
| 10 | Tartaric acid | 9.28 | 73, 147, 189, 219 | 3TMS | NIST | 85 | Water soluble |
| 11 | Asparagine | 9.36 | 73, 116, 147, 188, 232 | 3TBDMS | NIST | 87 | Water soluble |
| 12 | Aspartic acid | 9.485 | 73, 117, 147, 218, 232 | 3TMS | NIST | 88 | Water soluble |
| 13 | 5-Oxoproline | 9.545 | 73, 156, 230, 258 | 2TMS | NIST | 92 | Water soluble |
| 14 | 4-Aminobutanoic acid | 9.603 | 73, 147, 174, 304 | 3TMS | NIST | 88 | Water soluble |
| 15 | Glyceric acid | 9.777 | 73, 147, 189, 205, 292 | 3TMS | NIST | 96 | Water soluble |
| 16 | Linoleic acid | 9.973 | 67, 73, 129, 262, 337 | TMS | NIST | 85 | Lipid soluble |
| 17 | Eicosatrienoic acid | 10.02 | 67, 73, 117, 145, 192 | TMS | NIST | 91 | Lipid soluble |
| 18 | Oleic acid | 10.063 | 73, 117, 145, 339 | TMS | NIST | 84 | Lipid soluble |
| 19 | Stearic acid | 10.197 | 73, 117, 145, 341 | TMS | NIST | 94 | Lipid soluble |
| 20 | Glycerol | 10.517 | 73, 103, 147, 204 | 3TMS | NIST | 51 | Water soluble |
| 21 | Ribitol (IS) | 11.072 | 73, 103, 147, 217 | 5TMS | NIST | 93 | Water soluble |
| 22 | Tetracosane (IS) | 11.332 | 43, 57, 71, 85, 113 |  | NIST | 75 | Lipid soluble |
| 23 | Arachidic acid | 11.578 | 73, 117, 132, 369 | TMS | NIST | 80 | Lipid soluble |
| 24 | Fructose | 12.188 | 73, 103, 147, 217, 307 | 5TMS, Meox | NIST, STD | 78 | Water soluble |
| 25 | Glucose | 12.447 | 73, 117, 147, 189, 205, 319 | 5TMS, Meox | NIST, STD | 97 | Water soluble |
| 26 | Methyl galactoside | 13.153 | 73, 147, 204 | 4TMS | NIST | 97 | Water soluble |
| 27 | Palmitic Acid | 13.367 | 73, 75, 117, 313 | TMS | NIST | 93 | Water soluble |
| 28 | 1-Heptatriacotanol | 13.542 | 73, 81, 107, 147, 288 | TMS | NIST | 84 | Lipid soluble |
| 29 | Galactose | 13.565 | 73, 129, 147, 204 | 5TMS | NIST | 85 | Water soluble |
| 30 | Myo-Inositol | 13.605 | 73, 147, 217, 305 | 6TMS | NIST, STD | 97 | Water soluble |
| 31 | Glucitol | 13.843 | 73, 103, 147, 205, 319 | 6TMS | NIST | 42 | Water soluble |
| 32 | Galactaric acid | 15.170 | 73, 147, 292, 333 | 6TMS | NIST | 86 | Water soluble |
| 33 | Tocopherol | 15.832 | 73, 237, 502 | TMS | NIST, STD | 75 | Lipid soluble |
| 34 | 2-Ketoadipic acid | 15.955 | 73, 147, 245, 445 | 3TMS | NIST | 61 | Water soluble |
| 35 | Sucrose | 16.633 | 73, 147, 217, 361 | 8TMS | NIST, STD | 67 | Water soluble |
| 36 | 1-Hexacosanol | 18.175 | 73, 207, 439 | TMS | NIST | 71 | Water soluble |

^a^TMS, trimethylsilyl; Meox, methyloxime

^b^Metabolites were identified using library of the National Institute of Standards and Technology (NIST).
